# Assessment of Phenotypic Diversity in the USDA Collection of Quinoa Links Genotypic Adaptation to Germplasm Origin

**DOI:** 10.3390/plants11060738

**Published:** 2022-03-10

**Authors:** Muhammad Bilal Hafeez, Shahid Iqbal, Yuanyuan Li, Muhammad Sohail Saddiq, Shahzad M. A. Basra, Hui Zhang, Noreen Zahra, Muhammad Z. Akram, Daniel Bertero, Ramiro N. Curti

**Affiliations:** 1 Department of Agronomy, University of Agriculture, Faisalabad 38040, Pakistan; bilalhafeez32@gmail.com (M.B.H.); shehzadbasra@gmail.com (S.M.A.B.); zakram97@yahoo.com (M.Z.A.); 2 Department of Agronomy, Muhammad Nawaz Shareef, University of Agriculture, Multan 66000, Pakistan; sahu.shehdi@yahoo.com; 3 Instititute of Plant Breeding and Biotechnology, Muhammad Nawaz Shareef, University of Agriculture, Multan 66000, Pakistan; 4 Shandong Provincial Key Laboratory of Plant Stress Research, College of Life Science, Shandong Normal University, Jinan 250014, China; lyyzhh@gmail.com; 5 Department of Agronomy, Ghazi University, Dera Ghazi Khan 32200, Pakistan; sohail1540@gmail.com; 6 Department of Botany, University of Agriculture, Faisalabad 38040, Pakistan; noreenzahra59@gmail.com; 7 Cátedra de Producción Vegetal and Instituto de Investigaciones Fisiológicas y Ecológicas Vinculadas a la Agricultura (IFEVA)—CONICET, Facultad de Agronomía, Universidad de Buenos Aires, Buenos Aires C1417DSE, Argentina; bertero@agro.uba.ar; 8 Laboratorio de Investigaciones Botánicas (LABIBO), Facultad de Ciencias Naturales and Sede Regional Sur, Universidad Nacional de Salta—CCT-CONICET, Salta 4400, Argentina

**Keywords:** breeding, *Chenopodium quinoa* Willd., genetic structure, germplasm, heritability, effects of genotype by environment interaction

## Abstract

Quinoa’s germplasm evaluation is the first step towards determining its suitability under new environmental conditions. The aim of this study was to introduce suitable germplasm to the lowland areas of the Faisalabad Plain that could then be used to introduce quinoa more effectively to that region. A set of 117 quinoa genotypes belonging to the USDA quinoa collection was evaluated for 11 phenotypic quantitative traits (grain yield (Y), its biological and numerical components plus phenological variables) in a RCBD during two consecutive growing seasons at the University of Agriculture, Faisalabad, Pakistan under mid-autumn sowings. Genotypic performance changed across the years, however most phenotypic traits showed high heritability, from 0.75 for Harvest Index (HI) to 0.97 for aerial biomass (B) and Y. Ordination and cluster analyses differentiated four groups dominated by genotypes from: Peru and the Bolivian Highlands (G1); the Bolivian Highlands (G2); the Ballón collection (regarded as a cross between Bolivian and Sea Level (Chilean) genotypes) plus Bolivian Highlands (G3); and Ballón plus Sea Level (G4), this latter group being the most differentiated one. This genetic structure shared similarities with previous groups identified using SSR markers and G×E data from an international quinoa test. G4 genotypes showed the highest Y associated with higher B and seed numbers (SN), while HI made a significant contribution to yield determination in G2 and seed weight (SW) in G3. G1 and G2 showed the lowest Y associated with a lower B and SN. Moreover, SW showed a strongly negative association with SN in G2. Accordingly, G4 followed by G3 are better suited to the lowland areas of Faisalabad plain and the physiological traits underlying yield determination among genotypic groups should be considered in future breeding programs.

## 1. Introduction

The growing popularity of quinoa in the recent years made the crop require little introduction [1,2]. This is the consequence of sustained demand for its seed in the international market, particularly in the Health Food sector in developed countries [3]. Quinoa prices increased in a sustained way in the last few decades with a peak in 2014, reaching ~7000 US$ per ton [4]. This was accompanied by a parallel increased interest in quinoa evaluation and production in many countries outside the Andes, its traditional growing region [5,6]. It was not only its good nutritional balance and high prices, but also the capacity of the crop to face many limitations like water deficits, salinity, frost or poor soils that contributed to this interest [7,8]. Quinoa is currently being evaluated in all continents and commercial production is underway in a significant number of them [5,9,10]. Germplasm from different countries were evaluated and new breeding programs started in the US, Canada, the E.U., Australia, Israel, China, India and the Middle East, among others.

Germplasm evaluation, ideally involving contrasting origins when performed for the first time, is central to guide breeding and agronomic management [11,12]. For quinoa, the biggest undertaking was that organized by the FAO two decades ago [13] in which 24 genotypes with origins ranging from Colombia to Chile plus Europe were evaluated in locations in South America, Europe, Africa and Asia. This evaluation used pattern analysis and identified four genetic and four environmental groups based on yield performance. A strong degree of genotype by environment (G × E) interaction was detected demanding a clear characterization of the target environment and the identification of genotypes specifically suited to them. A similar analysis, this time including a more detailed analysis of the numerical (seed number and seed weight) and physiological (crop biomass and harvest index) components of yield was performed for local germplasm in N.W. Argentina by [14]. In addition to these analyses, where genotypic, environmental and G × E components of variation were identified, many more evaluations were performed with varying degree of detail in the analysis, such as in India [15], the Middle East and North Africa [16,17,18], Central Africa [19,20] and China [9]. More detailed descriptions of quinoa evaluation at national levels can be found in [2], in their report on the State of the Art of Quinoa in the World in 2013.

Besides these agronomic evaluations, quinoa genetic studies were approached using molecular markers. The first researcher to complete this work, [21], using allozyme markers, identified two distinct groups, from Central and Southern Chile and from the Central Andes, with a less clear distinction between germplasm from the Northern Andes (Colombia to Peru) and Southern Andes (Peru to Northwest Argentina and Northern Chile). A more recent evaluation [22] used microsatellite markers to characterize the USDA’s (United States Department of Agriculture) quinoa collection and was able to distinguish between Sea Level (quinoa originating or selected from accessions from low altitude environments in Central and Southern Chile [21]) and Andean types, plus a third group, named the Ballón collection according to the name of the germplasm donor to the USDA quinoa collection, which overlapped with both groups. This latter group originated in spontaneous crosses between Andean and Central Chile germplasm when multiplied at high field temperatures in New Mexico (E. Ballón pers. comm.). Using SNPs, Mizuno et al. [23] confirmed the structure of these three sub-groups (Northern and Southern Highlands plus Lowlands (central and southern Chile)).

Given the current legal restrictions to access germplasm from the Andean Region [24], the USDA germplasm collection (including more than 140 accessions) has been the basis of several adaptation and breeding programs internationally [15,16,17,18,20]. This collection includes accessions from most countries of traditional cultivation and provided sources of traits like male sterility [25] adaptations to hot environments [17,26,27], resistance to pre-harvest sprouting [28,29] or prolonged seed viability [30]. Based on this information, a better understanding of its agronomic performance is central and there are no published reports of it for this collection. Furthermore, as the Middle East is an area of recent fast expansion of quinoa cultivation, understanding quinoa agronomic behavior in these Mediterranean climates is also central in its own right.

The aim of this study was to screen a collection of quinoa genotypes for adaptation to yield under the low altitude environments of the Faisalabad region, Northern Pakistan. Specifically, we measured yield, its biological determinants and components plus phenology in 117 quinoa genotypes from the USDA germplasm collection to determine: (i) the relative contribution of genotype, environment and genotype-by-environment interaction effect to the phenotypic variation of those traits; (ii) the role of these traits in capturing the patterns of genotypic adaptation; (iii) the association between these patterns and the origin of materials; and (iv) the physiological trait combinations underlying yield-formation among genetic groups. Our working hypothesis is that the structure of phenotypic variation expressed in the patterns of performance of quinoa in Pakistan reflects the geographical origin of the materials.

## 2. Material and Methods

### 2.1. Plant Genetic Materials and Field Experiments

One hundred-seventeen quinoa genotypes belonging to the USDA-NPGS (United States Department of Agriculture) collection were utilized in this study (Table S1). A two-years field study was conducted through a randomized complete block design (RCBD) with three replications per genotype and year in order to evaluate the performance of this collection. The crop was sown on 21 November and 20 November in 2016 and 2017, respectively, at the Agronomy Farm (73.88° E, 31.88° N; elevation 184 masl) of the University of Agriculture of Faisalabad (UAF), Pakistan. Daily recorded values of air temperature and rainfall events at the experimental site were obtained from the Agricultural Meteorology Cell, UAF. For each experiment, the site soil was ploughed to 30 cm depth and subsequently harrowed. Ridges, 30 cm in height, were prepared at 75 cm spacing. Seeds were sown on the top of each ridge at 15 cm spacing with a hand dibble method. Each experimental plot comprised two ridges with a length of 3 m. Two terminal quinoa plants at each end of the ridge were used as guard plants, giving a net plot size of two ridges of 2.75 m in length. For fertilization, nitrogen (N), phosphorus (P) and potassium (K) were applied at 75, 60, and 60 kg ha^−1^, respectively, during both years. Fertilizers used were urea (46% N), diammonium phosphate (18% N, 46% P_2_O_5_) and sulfate of potassium (50% K_2_O). A half dose of N and a full dose of P and K were applied as a basal dose at sowing and the remaining N was added 75 days after sowing. During the entire crop period, four irrigations of 330 mm total were applied including the pre-sowing irrigation, recommended as optimal for the cultivation of quinoa, for the conditions of Pakistan [31]. The field was kept free from weeds by hand hoeing, whereas no insecticide/pesticide and herbicide were used throughout both experimental years.

### 2.2. Measurements

A set of 11 phenotypic traits encompassing grain yield-related attributes and crop phenological stages were obtained for each plot. Grain yield (Y, g m^−2^) and its biological determinants aerial biomass (B, g m^−2^), harvest index (HI) plus terminal panicle grain yield (Pan Y, g m^−2^), and the numerical components seed number (SN, # m^−2^) and weight (SW, mg) were obtained from five uniform tagged plants from the middle of each row, avoiding border plants. Plants were harvested with a sickle, sundried for three to five days and later on threshed manually to measure yield attributes using a digital scale. HI was estimated as the Y/B ratio. SN was estimated considering the final harvest data as the ratio of Y to the average individual SW. Individual SW was estimated by manually counting and weighing 1000 grains in each replicate plot. In addition, for the five plants tagged in each plot, the plant height (Ht, cm) and terminal panicle length (Pan Ht, cm) were measured with a ruler. Ht was measured from the soil to the top of the terminal panicle, whereas the Pan Ht was measured between the basal and the last node below the main panicle.

Crop development stages (recorded when at least 50% of the plants in each plot had reached the stage) were determined as emergence, first anthesis (extrusion of anthers completed), and physiological maturity (when grain could not be nail dented). Thus, we defined the main phenological phases as the emergence–anthesis (E–Ant) and anthesis-physiological maturity (Ant–PM) periods. Besides that, the crop duration (Cycle) period was recorded as the time between emergence and harvest.

### 2.3. Data Analysis

Linear mixed models were set up to examine the relative contribution of genotype and genotype-by-environment interaction effects to the phenotypic variation of all phenotypic traits across experiments. The phenotypic observation $y_{ijk}$ on genotype *i* in block *k* of environment *j* was modelled following the expression:

$$y_{ijk}=\mu +G_{i}+E_{j}+{\left(GE\right)}_{ij}+B_{k\left(j\right)}+\varepsilon_{ijk}$$

with μ designating the general intercept, *G_i_~N* (0, σ^2^_g_) is the random main effect of the *i*-th genotype, $E_{j}$ is the fixed main effect of the *j*-th environment (years), *GE_ij_~N* (0, σ^2^_ge_) is the random interaction effect of the *i*-th genotype and the *j*-th environment, *B_k(j)_~N* (0, σ^2^_b_)> is the random effect of the *k*-th block nested within the environment *j*-th, and $\varepsilon_{ijk}$ is the residual plot error associated with the observation $y_{ijk}$. All linear mixed models were fitted with the function gamen_met of the R package metan (multi-environment trial analysis) [32]. This function estimates the variance components of random effects by Restricted Maximum Likelihood (REML), whereas their significance by a likelihood ratio (LRT) test, comparing a full model with all random terms with each other, without one of the random terms (reduced model). In addition, broad-sense heritability (H) was computed for all traits.

To determine the role of phenotypic traits in capturing similarities in genotypic response, Pattern Analysis (PA), defined as the combined application of ordination and classification multivariate analyses (principal component analysis (PCA) and Cluster Analysis (CA)), was used. This set of analyses was based on predictions computed from random terms obtained from REML analyses (see above). First, we computed the Best Linear Unbiased Predictors (BLUPs) for genotype, and genotype-by environment interaction terms, and then the predictions adjusted by the BLUPs’ effects were used to build an array of 117 genotypes × 11 traits. Previous to PA, the array was trait-standardized by removing the traits’ grand mean and dividing the remainder by the within-trait standard deviation. For classification (CA), the hierarchical agglomerative method, with the incremental sum of squares as fusion criteria [33], was chosen, using the Euclidean distance. A dendrogram of genotypes was produced in order to investigate the grouping of genotypes according to the evaluated phenotypic traits. The optimal number of clusters was defined according to 30 indices computed from the NbClust package [34]. Then, the results of the dendrogram obtained were compared with the genetic diversity study of [22], to investigate the relatedness between the patterns of genotypic responses and the origin of materials. In addition, we tested the differences among groups for all agronomic traits by means of analyses of variance. Mean comparisons were based on Tukey’s HSD test.

For ordination, a PCA was performed using the singular value decomposition algorithm on the Euclidean standardized distance of the two-way array of genotypes × traits, using the FactoMineR package [35]. In order to investigate the interrelations between genotypes and traits, a Biplot of the first and second axis was obtained. In the Biplot the symbols (genotypes) were depicted according to the dendrogram results obtained from CA. To study the physiological traits combinations underlying the yield-formation among genetic groups, we approximated the correlations between grain yield components by inspecting the angles formed between vectors (traits) in the Biplot. Rules of interpretation according to Biplot properties are: angles below 90° approximate a positive association between vectors; angles above 90° approximate a negative association; and angles at 90° indicate no association. All statistical analyses were performed within the R environment version 4.0.5 (2021).

## 3. Results

### 3.1. Effects of Growing Conditions on Phenotypic Variation

Growing conditions in Faisalabad, Pakistan were similar among the experimental years, but with a lower minimum temperature and rainfall during the second season (Table 1). Climatic conditions of these two particular years matched the general climate patterns for the same locality as corroborated by comparison with weather data from the years 1981–2015 in the NASA website (https://power.larc.nasa.gov/data-access-viewer/, accessed on 15 October 2021). The seasonal photoperiod varied from 10.4 to 12.9 h day^−1^ for the crop cycle, with most genotypes flowering during spring and maturing in summer (i.e., under high photo-thermal conditions). The environmental means for phenotypic traits, i.e., the average across genotypes, differed between growing seasons for Pan Ht, B, Pan Y, SW, E–Ant and Cycle, with a trend towards higher values in the second growing season (Table 1).

### 3.2. Variance Components and Heritability

Estimated components of variance and their relative contribution to phenotypic variation are shown in Table 2. The G effect contributed significantly to phenotypic variation in all traits, whereas the G × E effect also contributed significantly to phenotypic variation save for Pan Ht, B, Pan Y and Y (Table 2). The G term accounted for a larger proportion of variation than the G × E term for phenotypic traits, in which both sources of variation were significant (with G/G × E ranges from 5.1 (SW) to 25.3 (SN)). Consequently, high estimates of broad-sense heritability were observed (ranging from 0.82 for SW to 0.97 for B and Y, respectively; Table 2).

### 3.3. Phenotypic Variation Patterns in the USDA Germplasm Collection

**Figure 1:**
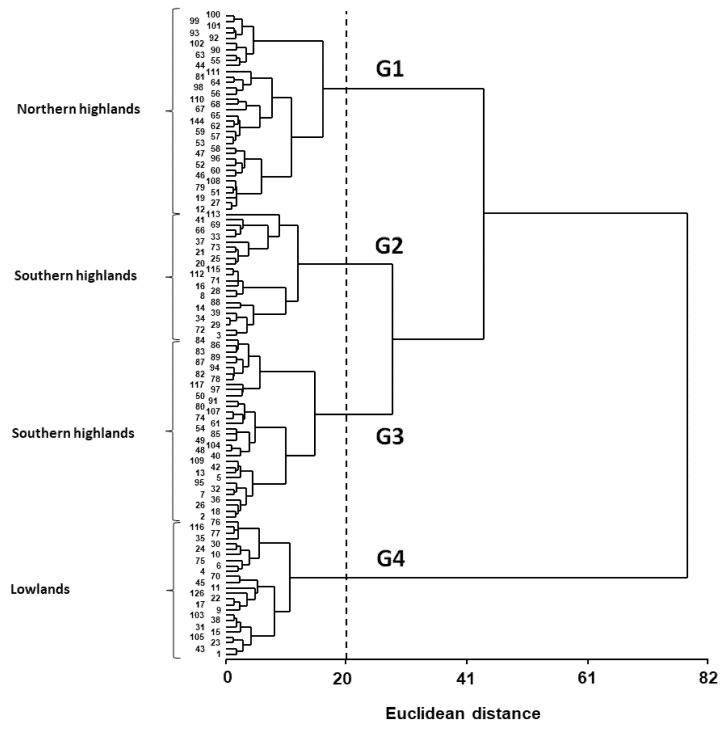
Dendrogram showing 117 genotypes of quinoa grouped according to Ward cluster analysis using 11 phenotypic traits. To the left of the dendrogram the groups are named according to the ecotype classification based on microsatellite markers by [22]. To the right of the dendrogram the groups are named according to the classification obtained in the present study; G1: genotypes from Peruvian and Bolivian highlands, G2: genotypes from Bolivian highlands, G3: genotypes from Ballón collection and Bolivian highlands and G4: genotypes from Ballón collection and Sea Level. See Table S1 for more information about genotypes codes and both classifications.

The 117 quinoa genotypes clustered into four clearly distinct groups (Figure 1). Group 1 (G1) consisted of 35 entries dominated by genotypes from the Bolivian (17) and Peruvian (12) Highlands plus one accession from the Peruvian Inter-Andean Valleys (Rosa de Junín, genotype 68), three from the Ballón collection (genotypes 12, 19 and 44), one from Sea Level (genotype 108), and one from an unknown origin (genotype 110) (Figure 1 and Table S1). Most of these genotypes belong to the Andean group according to [22] and were classified in the northern highland subgroup based on microsatellite’s markers (Table S1). Genotypes from this group showed the lowest values for Y, B, HI, SN and SW, but the highest values for traits such as Ant–PM and Cycle (Table 3). Group 2 (G2) consisted of 22 entries with most genotypes from the Bolivian Highlands (16) plus three accessions from Sea Level (genotypes 74, 104 and 107), two from Peru (genotypes 54 and 61) and one from the Ballón collection (genotype 40) (Figure 1 and Table S1). Most of these genotypes belong to the Andean group and were classified in the southern highland subgroup based on microsatellites’ markers (Table S1). Genotypes from this group showed the lowest values for most phenotypic traits, but higher values for Y, B, HI, SN and SW compared with G1 (Table 3). Group 3 (G3) consisted of 31 entries dominated by genotypes from the Ballón collection (21) and the Bolivian Highlands (5) plus three accessions from Northwest Argentina (genotypes 67, 71 and 72) and two from Sea Level (genotypes 73 and 109) (Figure 1 and Table S1). Most of these genotypes were classified in the lowland and southern highland subgroup based on microsatellite’s markers (Table S1). Genotypes from this group showed higher values for most phenotypic traits compared with G1–G2 and the highest SW within the evaluated collection (Table 3). Group 4 (G4) was composed of 29 entries dominated by genotypes from the Ballón collection (17) and Sea Level (9) plus the Isluga (genotype 23), Cochabamba (genotype 69) and Plant Virus (genotype 43) (Figure 1 and Table S1). Most of these genotypes were classified in the lowland group based on microsatellites’ markers (Table S1). This group showed the highest values for most phenotypic traits, except for traits such as SW, Ant–PM and Cycle (Table 3).

**Figure 2:**
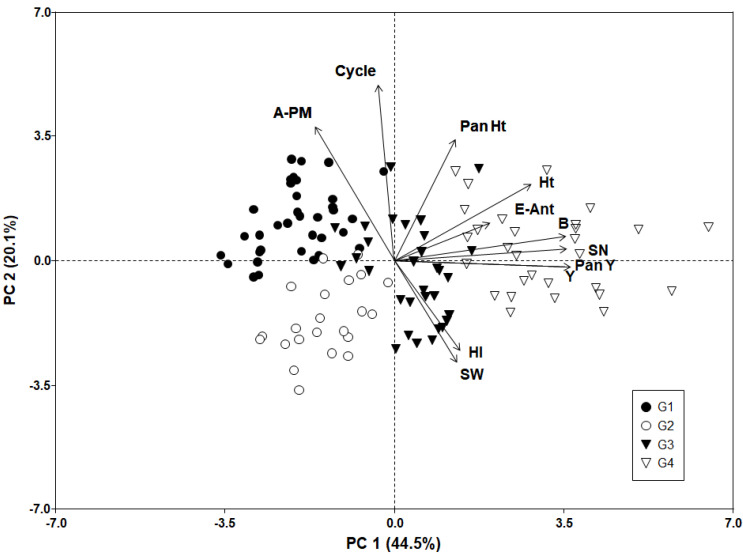
Biplot of the 1st and 2nd principal components for 117 accessions of quinoa described by 11 phenotypic traits. Genotypes are represented by symbols and traits by vectors. Same color symbols indicate genotype groups with members of a similar response pattern. G1: Peruvian and Bolivian Highlands; G2: Bolivian highlands; G3: Ballón collection and Bolivian highlands; and G4: Ballón collection and Sea Level.

Results of the ordination analyses are displayed in the Biplot of the 1st and 2nd principal components, which, when combined, accounted for ~65% of total variation (Figure 2). The trait vectors covered a wide range of Euclidean space suggesting a strong contrast among the phenotypic traits evaluated. The angle between grain yield (Y) and its biological determinants and components (B, SN, Pan Y, HI and SW) plus E–Ant, is smaller than 90° (Figure 2), which suggests that most of these traits are positively associated within the collection evaluated. Traits such as HI and SW are positively correlated, but both lack association with Ht as their angles are close to 90° (Figure 2); whereas strong and negative associations (angles larger than 90°) were found between Y plus its related traits (B, SN, Pan Y, HI and SW) and Ant–PM plus Cycle (Figure 2). In turn, the E–Ant phase lacks association with Ant–PM duration as their angles are close to 90° (Figure 2).

The 1st principal component (PC1) explained 44.5% of the total variation and ordered the genotypes according to Y, its related traits (B, SN, Pan Y, HI, and SW) and time to anthesis. As indicated by Figure 2, genotypes with higher Y, B, HI, SN, Pan Y, Ht, and E–Ant duration were placed to the right of the Biplot. Most of these genotypes are from G4 (Ballón collection plus Sea Level) and represent the most differentiated group (Figure 1 and Figure 2). Genotypes to the left side of the PC1 showed lower values for Y and its related traits plus time to anthesis (Table 3 and Figure 2). Most of them are from G1 and G2 (Peru and Bolivian Highlands) (Figure 2 and Table S1). Genotypes from G3 (Ballón collection plus Bolivian Highlands) are located in an intermediate place on PC1 (Figure 2). They are characterized by the most Y and its related traits, similar to those from G4 and to some extent to G2 (mainly by HI, SW, and Cycle), but by Pan Ht (terminal panicle length) to those from G1 (Table 3 and Figure 2).

The second principal component (PC2) explained 20.1% of the total variation and accounted for the effects of the contrasting traits Ant–PM, Pan Ht, Cycle, and HI plus SW, emphasizing the differences between groups G1 and G2 (Figure 2). G1 tended to be at the top left-hand quadrant of the Biplot, which indicated that it had high values for Pan Ht, Ant–PM, and Cycle, but low values for HI plus SW and included the genotypes with the longest duration within the collection (Figure 2 and Table 3). The genotypes from G2 showed contrasting values for most of those phenotypic traits and are placed toward the bottom left-hand quadrant of the Biplot (Figure 2), showing high values for HI and SW, but lower values for Pan Ht, Ant–PM, and Cycle, and could be considered as the genotypic set that was the most precocious within the collection (Table 3).

## 4. Discussion

The results of this study show the first phenotyping assessment carried out on a comprehensive set of quinoa accessions from the USDA germplasm collection accounting for yield and its components. This collection was the basis of several adaptations and breeding programs internationally and, as such, made a highly significant contribution to the global expansion of this crop [17,36,37,38,39,40,41,42]. According to our results, the environment contributed to phenotypic variation in most traits (Table 1); however, their magnitude was relatively low compared with contributions of G and G × E effects. This is surprising as usually the E component accounts for the highest proportion of variation, also for quinoa [13,14,15] and is explained by its similarity between both evaluation years (which reflected general climate patterns for the region as mentioned). Consequently, the G effects, accounting for a large proportion of variation and its contribution, was relatively high compared to G × E effects for most traits (Table 2). These results match those found under Tropical and Mediterranean conditions involving subsets of genotypes originating from the USDA collection [15,41]. Accordingly, the overall picture arising from this comparison highlights the major role of G effects in determining phenotypic values in a large set of representative genotypes from that collection.

The high heritability observed for all traits implies that phenotypic variation reflects the patterns of genotypic adaptation. The genotypic groups found in the present study clearly showed differences in their performance (Table 3). Phenotypic trait combinations observed in genotypes from G3 and G4 determined their higher suitability for cultivation under the Mediterranean conditions of Faisalabad. This set of genotypes with the highest values for yield and its related traits showed intermediate crop cycle durations (Table 3). Conversely, genotypes from G1 and G2 with the lowest values for most traits showed either a longer (G1) or shorter (G2) crop cycle, respectively (Table 3). This pattern of response was observed in other evaluations conducted at high latitudes or under tropical and Mediterranean conditions [15,43,44]. Early or late-maturing genotypes, in general, have been shown to perform better within a narrow range of environments, whereas genotypes with an intermediate crop cycle are better adapted to a broader range of conditions [13,14,45,46]. Genotypic variations in sensitivity to temperature and photoperiod conditions are the main factors controlling phenology and explains this contrast in genotypic adaptation in quinoa [47,48].

The hierarchical agglomerative groupings identified four genotypic groups associated with the genotype’s environments of origin (Figure 1). This grouping shows a close correspondence with the proposed quinoa genetic groups based on molecular studies [22,23], which distinguished three groups of accessions, namely Lowland, Northern Highlands and Southern Highlands, corresponding to G4, G1 and G2–G3 of this study, respectively (Table S1). Moreover, the four genotypic groups found here partially resemble those found in early evaluations conducted using a large set of quinoa cultivars representing all environments of origin of the crop [13] or on a local basis with germplasm from N.W. Argentina [14]. The difference between this classification and those obtained on the basis of yield performance was observed regarding the genotypic composition among groups. Within G1, accessions from the northern highlands dominated, however southern highlands’ accessions were also represented (Table S1). Besides, while accessions from the southern highlands were distributed among G2 and G3, they clearly dominated in G2 (Table S1). These results contrast with the clear distinction between northern- and southern highlands’ types found by [13]. The highland accessions from the N.W Argentina germplasm were grouped with accessions belonging to the Ballón collection in G3 (Table S1), while it was expected that they would be grouped with accessions from G2. On the other hand, a correspondence was observed between accessions included in G4 and the sea-level type as in [13,47].

The genotypes originating from the Ballón collection have been little evaluated in other studies [2,15,17,49], and were represented with at least one accession in all groups, and clearly dominated in G3 and G4 (Figure 1 and Table S1). This grouping pattern reinforces the notion of an inter-regional origin for the Ballón genotypes arising from spontaneous crosses between genotypes from southern Andean Highlands (G3) and Sea Level (G4) groups (E. Ballón pers. com.) [22]. Genotypic adaptation in quinoa has largely been related to the environment of the origin of the materials. Thus, several early and recent introduction programs outside the Andean region were frequently based on accessions originating from Sea Level environments or from genotypes of that origin [43,44,46]. The results presented here are in line with the above statements, as genotypes originating from Sea Level were the best performers in the Faisalabad environments (Table 3 and Figure 2). However, genotypes from the Ballón collection also showed phenotypic traits’ combinations that were desirable for cultivation in those sites and deserve further exploration in future studies. An example of this is accession AMES 13,737 (2 Want) which exhibited good performance in Central Argentina [50,51] and the heat-stressed conditions of UAE [17].

The physiological traits’ combinations underlying yield-formation differed among the four genetic groups and resembled results from other studies [13,14]. High aerial biomass and a long time to anthesis were suitable traits’ combinations for determining higher grain yield associated with a higher seed number (Figure 2). This set of phenotypic traits was observed in genotypes from G4 and to lesser extent from G3 (Figure 2). In addition, both genetic groups showed medium crop cycle duration (Table 3). A longer or shorter time for seed-filling duration determines poor performance as the genotypes with the longest time (G1) or the earliest (G2) showed a lower grain yield (Figure 2 and Table 3). However, early-maturing genotypes from G2 and G3 improved grain yield and seed weight associated with increases in harvest index (Figure 2). These different combinations of underlying physiological traits determining cultivars with similar grain yield performance have been observed in several major crops [52,53,54,55], and also in quinoa [14]. In addition, the lack of association found between the developmental phases of E–Ant and Ant–PM (Figure 2), implies that there is scope for manipulating the plant’s developmental duration through breeding targeted to obtain genotypes with different duration combinations by crossing accessions from groups showing contrasting developmental phase durations as proposed by [56], and also observed in crops like soybean [57]. In this sense, future breeding programs aimed to increase grain yield in quinoa under Faisalabad cultivation conditions and similar environments under Mediterranean Climates could be targeted by combining phenotypic traits found in genotypes from groups G3 and G4, or by exploiting indirect selection for phenotypic traits determining yield across both groups, respectively. Finally, more testing sites are needed across countries from North Africa and East Asia, which share similarities in the Mediterranean conditions with Faisalabad, to evaluate the degree of repeatability in the genotypic response patterns observed in the present study.

## 5. Conclusions

The performance (average yields) of quinoa under Pakistan’s Mediterranean conditions was poorer than evaluations carried out in locations with similar climate regimes [17,27,58,59], with only G4 reaching agronomic significance. However, the patterns of genotypic adaptation were reflected by the strong genotype effect found in all yields and its related traits, whereas the patterns of variation matched the environments of origin of materials. Furthermore, the physiological traits combinations underlying yield-formation varied among the genotypic groups, determining differences in adaptation to yield. The genotypes originating from Sea Level, the Ballón collection and the Bolivian Highlands (G3 and G4) show a suitable set of phenotypic traits able to expand quinoa cultivation under the low altitude environments of the Faisalabad region. In addition, seminal breeding programs established with the prospect to advance the adaptation of quinoa to these conditions could benefit from exploiting indirect selection for traits contributing to yield generation among genotypes from those groups.

## Figures and Tables

**Table 1:** Growing conditions in Faisalabad and their effect on measured phenotypic traits. Maximum, minimum and mean temperature are ranges of monthly values whereas rainfall are totals for the growing season (November–April). For phenotypic traits the values are shown as means ± SE.

	Season		
	2016	2017	
Max. temp. (°C)	17.6–37.7	21.5–36.8	(27.3–35.4) ^a^
Min. temp. (°C)	8.2–20.9	5.5–20.8	(2.2–13.0) ^a^
Mean temp. (°C)	12.9–29.3	13.5–28.8	(12.5–27.7) ^a^
Rainfall (mm)	60	36	(24.4) ^a^
Ht (cm) ^b^	94.3 ± 1.6	97.6 ± 1.8 **	
Pan Ht (cm)	29.3 ± 0.5	29.4 ± 0.4	
B (g m^−2^)	67.9 ± 3.2	71.0 ± 3.1 *	
HI	35.4 ± 0.6	35.7 ± 0.6	
Pan Y (g m^−2^)	17.0 ± 0.9	18.3 ± 0.9 **	
Y (g m^−2^)	24.6 ± 1.3	25.6 ± 1.3	
SN (# m^−2^)	7.7 × 10^6^ ± 4 × 10^5^	7.9 × 10^6^ ± 3.9 × 10^5^	
SW (mg)	3.1 × 10^−3^ ± 3.6 × 10^−5^	3.2 × 10^−3^ ± 4 × 10^−5^ **	
E–-Ant (days)	64.7 ± 0.3	67.9 ± 0.3 **	
Ant–PM (days)	51.3 ± 0.5	50.6 ± 0.5	
Cycle (days)	129.6 ± 0.5	134.9 ± 0.4 **	

** significant at *p* < 0.01, * significant at *p* < 0.05. ^a^ Range values correspond to the historical weather records during the last 35 years (from 1981 to 2015) downloaded from the NASA website (https://power.larc.nasa.gov/data-access-viewer/, accessed on 15 October 2021); ^b^ Abbreviations: Ht: plant height, Pan Ht: terminal panicle length, B: aerial biomass, HI: harvest index, Pan Y: terminal panicle grain yield: Y: yield, SN: seed number, SW: seed weight, E–Ant: emergence-anthesis period: Ant–PM: anthesis–physiological maturity period, and Cycle: crop duration.

**Table 2:** Relative contribution of estimated variance components and broad-sense heritability (H) for 11 phenotypic traits measured in 117 genotypes of quinoa across two years in Faisalabad.

Trait	σ^2^_g_	σ^2^_ge_	σ^2^_g_/σ^2^_ge_	H
Ht	942.0 **	46.9 **	20.1	0.90
Pan Ht	60.1 **	0.15 ns		0.87
B	3364.0 **	7.8 ns		0.97
HI	89.5 **	11.0 **	8.1	0.75
Pan Y	17.7 *	15.9 ns		0.96
Y	57.3 **	32.2 ns		0.97
SN	5.2 × 10^13^ **	2.0 ×10^12^ **	25.3	0.93
SW	4.3 × 10^−7^ **	8.4 × 10^−8^ **	5.1	0.82
E–Ant	35.1 **	4.0 **	8.8	0.87
Ant–PM	78.8 **	7.2 **	10.9	0.90
Cycle	63.8 **	5.2 **	12.3	0.92

** significant at *p* < 0.01, * significant at *p* < 0.05, ^ns^ non-significant.

**Table 3:** Agronomic traits of the four genotype groups resulting from a hierarchical agglomerative clustering method.

Group	Ht ^a^ (cm)	Pan Ht (cm)	B (g m^−2^)	HI	Pan Y (g m^−2^)	Y (g m^−2^)	SN (# m^−2^)	SW (mg)	E–Ant (days)	Ant–PM (days)	Cycle (days)
G1	79.7 a	29.8 b	26.2 a	0.31 a	5.0 a	6.6 a	2.8 × 10^6^ a	2.6 a	63 a	60 b	138 a
G2	67.9 a	25.0 a	26.0 a	0.37 bc	7.7 a	9.9 a	3.2 ×10^6^ a	3.3 b	63 a	45 a	123 c
G3	110.5 b	29.3 b	73.4 b	0.35 b	17.0 b	23.8 b	6.6 × 10^6^ b	3.6 b	69 b	46 a	131 b
G4	121.4 b	32.3 b	150.5 c	0.40 c	41.2 c	60.4 c	1.9 × 10^7^ c	3.3 b	69 b	49 a	133 b

^a^ Abbreviations: Ht: plant height; Pan Ht: terminal panicle length; B: aerial biomass; HI: harvest index (%); Pan Y: terminal panicle grain yield; Y: yield; SN: seed number; SW: seed weight; E–Ant: emergence–anthesis period; Ant–PM: anthesis–physiological maturity period; Cycle: crop duration. Different letters following agronomic values indicate significant differences based on Tukey’s HSD test.

## Data Availability

The data presented in this study are available on request from the corresponding author. The data are not publicly available due to data protection for varietal development purposes.

## Supplementary Materials

The following supporting information can be downloaded at: https://www.mdpi.com/article/10.3390/plants11060738/s1, Table S1: Passport data on quinoa germplasm evaluated under Faisalabad environmental conditions.
