# Supplementary material for: Assessment of Phenotypic Diversity in the USDA Collection of Quinoa Links Genotypic Adaptation to Germplasm Origin
Source: Plants (Basel). 2022 Mar 10;11(6):738. doi: 10.3390/plants11060738 (PMC8954766; doi:10.3390/plants11060738)
Supplement: Supplementary file 1 [file plants-11-00738-s001.zip › plants-1596393-supplementary.pdf]

Table S1. Passport data on quinoa germplasm evaluated under Faisalabad environmental conditions.

Genotype code	Accession No.	Accession name	Passport/origin[a]	Ecotype group[b]	Genotypic group[c]
1	Ames 13721*	90R	USA, New Mexico	Lowland	4
2	Ames 13722*	87R	USA, New Mexico	Lowland	3
3	Ames 13723*	81R	USA, New Mexico	Lowland	3
4	Ames 13724*	18GR	USA, New Mexico	Lowland	4
5	Ames 13725*	46TES	USA, New Mexico		3
6	Ames 13726*	49ALC	USA, New Mexico	Lowland	4
7	Ames 13727*	38TES	USA, New Mexico	Lowland	3
8	Ames 13728*	27GR	USA, New Mexico	Lowland	3
9	Ames 13729*	23P	USA, New Mexico	Lowland	4
10	Ames 13730*	V2	USA, New Mexico	Lowland	4
11	Ames 13731*	42TES	USA, New Mexico	Lowland	4
12	Ames 13732*	40TES	USA, New Mexico		1
13	Ames 13733*	20TES	USA, New Mexico	Southern Highland	3
14	Ames 13734*	47TES	USA, New Mexico	Lowland	3
15	Ames 13735*	17GR	USA, New Mexico	Lowland	4
16	Ames 13736*	30TES	USA, New Mexico	Lowland	3
17	Ames 13737*	V7	USA, New Mexico	Lowland	4
18	Ames 13738*	20TES	USA, New Mexico	Lowland	3
19	Ames 13739*	29TES	USA, New Mexico	Southern Highland	1
20	Ames 13740*	50ALC	USA, New Mexico	Lowland	3
21	Ames 13741*	54ALC	USA, New Mexico	Lowland	3
22	Ames 13742*	6P	USA, New Mexico	Lowland	4
23	Ames 13743*	ISLUGA	Chile	Lowland	4
24	Ames 13744*	409	USA, New Mexico	Lowland	4
25	Ames 13745*	KASLAEA	USA, New Mexico	Lowland	3
26	Ames 13746*	PISON	USA, New Mexico	Lowland	3
27	Ames 13747*	APELAWA	Bolivia	Lowland	1
28	Ames 13748*	COPACABANA	USA, New Mexico	Lowland	3
29	Ames 13749*	41P	USA, New Mexico	Lowland	3
30	Ames 13750*	A5P	USA, New Mexico	Lowland	4
31	Ames 13751*	42P	USA, New Mexico	Lowland	4
32	Ames 13752*	62P	USA, New Mexico		3
33	Ames 13753*	70P	USA, New Mexico	Lowland	4
34	Ames 13754*	52ALC	USA, New Mexico	Lowland	3
35	Ames 13755*	43ALC	USA, New Mexico	Lowland	4
36	Ames 13756*	3UISE	USA, New Mexico	Lowland	3
37	Ames 13757*	53ALC	USA, New Mexico	Lowland	3
38	Ames 13758*	98R	USA, New Mexico	Lowland	4
39	Ames 13759*	71P	USA, New Mexico	Lowland	3
40	Ames 13760*	75P	USA, New Mexico	Lowland	2
41	Ames 13761*	47TES	USA, New Mexico	Lowland	4
42	Ames 13762*	47TES	USA, New Mexico	Lowland	3
43	NSL 91567	PLANT VIRUS	USA, New Mexico	Lowland	4
44	NSL 92331*	JAPANESE STRAIN	USA, Washington	Lowland	1
45	PI 433232	-	Chile, Groben	Lowland	4
46	PI 470932	PASANRALLE	Bolivia	Southern Highland	1

47	PI 478410	R-66	Bolivia, La Paz	Northern Highland	1
48	PI 478411	R-67	Bolivia, La Paz	Northern Highland	2
49	PI 478414	R-70	Bolivia, La Paz	Northern Highland	2
50	PI 478415	R-71	Bolivia, La Paz	Southern Highland	2
51	PI 478418	R-132	Bolivia, Potosi	Northern Highland	1
52	PI 510534	Mezclada Tres Variedades (Spain)	Peru	Northern Highland	1
53	PI 510536	Quinoa Negra (Spain)	Peru	Northern Highland	1
54	PI 510537	Koito Jaira (Aymara), Quinoa Plomo Or Qu	Peru	Northern Highland	2
55	PI 510538	Jaro Juira (Aymara), Quinoa Amarga (Spain)	Peru	Northern Highland	1
56	PI 510540	JachaNaka Jaro Joira (Aymara), Quinoa A	Peru	Southern Highland	1
57	PI 510541	JachaNakaJancco Jorra (Aymara), Quinoa	Peru	Northern Highland	1
58	PI 510542	Villa Juira (Aymara), Quinoa Rojo (Spain)	Peru	Northern Highland	1
59	PI 510543	KelloJuira (Aymara), Quinoa Amarillo (Spain)	Peru	Northern Highland	1
60	PI 510545	Ccankolla (Aymara), Quinoa Sajama Jusi	Peru	Southern Highland	1
61	PI 510546	JanccoJuira (Aymara), Quinoa Blanca (Spain)	Peru	Northern Highland	2
62	PI 510547	Ara Juira (Aymara), Quinoa Silvestre (Spain)	Peru	Northern Highland	1
63	PI 510549	Yulaj K'oyto (Quechua), Quinoa Plomo (Spain)	Peru	Northern Highland	1
64	PI 510550	Q'elloQuinoa (Quechua), Quinoa Amarilla	Peru	Northern Highland	1
65	PI 510551	Quinoa (Quechua), QuinoaVar. K'anko'lla	Peru	Northern Highland	1
66	PI 584524	QQ056	Chile	Lowland	4
67	PI 587173	LP128	Argentina, Jujuy	Northern Highland	3
68	PI 596498	Rosa Junin	Peru, Cuzco	Northern Highland	1
69	PI 614002	Ames 10334	Bolivia, Cochabamba	Northern Highland	4
70	PI 614880	QQ065	Chile, Los Lagos	Lowland	4
71	PI 614881	QQ95	Argentina, Jujuy	Northern Highland	3
72	PI 614884	QQ87	Argentina, Jujuy	Southern Highland	3
73	PI 614885	QQ57	Chile, Bio-Bio	Lowland	3
74	PI 614886	QQ74	Chile, Maule	Lowland	2
75	PI 614887	QQ63	Chile, Bio-Bio	Lowland	4
76	PI 614888	QQ61	Chile, Bio-Bio	Lowland	4
77	PI 614889	QQ59	Chile, Bio-Bio	Lowland	4
78	PI 614901	CQ101	Bolivia, Oruro	Southern Highland	2
79	PI 614903	CQ103	Bolivia, Oruro	Southern Highland	1
80	PI 614906	CQ106	Bolivia, Oruro	Southern Highland	2
81	PI 614907	CQ107	Bolivia, Oruro	Southern Highland	1
82	PI 614908	CQ108	Bolivia, Oruro	Southern Highland	2
83	PI 614911	CQ111	Bolivia, Oruro	Southern Highland	2
84	PI 614912	CQ 112	Bolivia, Oruro	Southern Highland	2
85	PI 614915	CQ115	Bolivia, Oruro	Southern Highland	2
86	PI 614917	CQ117	Bolivia, Oruro	Southern Highland	2
87	PI 614918	CQ118	Bolivia, Oruro	Southern Highland	2
88	PI 614919	CQ119	Bolivia, Oruro	Southern Highland	3
89	PI 614920	CQ120	Bolivia, Oruro	Southern Highland	2
90	PI 614921	CQ121	Bolivia, Oruro	Southern Highland	1
91	PI 614922	Sayaña	Bolivia, La Paz	Northern Highland	2
92	PI 614924	CQ124	Bolivia, La Paz	Northern Highland	1
93	PI 614925	CQ125	Bolivia, La Paz	Northern Highland	1
94	PI 614926	CQ126	Bolivia, La Paz	Northern Highland	2
95	PI 614927	CQ127	Bolivia, La Paz	Southern Highland	3

96	PI 614928	CQ128	Bolivia, La Paz	Southern Highland	1
97	PI 614930	CQ130	Bolivia, La Paz	Southern Highland	2
98	PI 614932	CQ132	Bolivia, Oruro	Southern Highland	1
99	PI 614934	CQ134	Bolivia, Oruro	Southern Highland	1
100	PI 614935	CQ135	Bolivia, Oruro	Southern Highland	1
101	PI 614937	CQ138	Bolivia, Oruro	Northern Highland	1
102	PI 614938	CQ139	Bolivia, Oruro	Northern Highland	1
103	PI 634917	Pichilemu	Chile	Lowland	4
104	PI 634918	Baer	Chile	Lowland	2
105	PI 634919	Pichaman	Chile	Lowland	4
106	PI 634921	UDEC-2	Chile	Lowland	4
107	PI 634922	UDEC-4	Chile	Lowland	2
108	PI 634923	UDEC-1	Chile	Lowland	1
109	PI 634925	UDEC-3	Chile	Lowland	3
110	PI 665272	Bianra De Juny	Australia	Northern Highland	1
111	PI 665273	Line 2-31	Bolivia, La Paz	Northern Highland	1
112	PI 665274	Line 0291	Bolivia, La Paz	Northern Highland	3
113	PI 665275	Line 0692	Bolivia, La Paz	Northern Highland	3
114	PI 665276	Line 1376	Bolivia, La Paz	Northern Highland	1
115	PI 665277	Line 1599	Bolivia, La Paz	Northern Highland	3
116	PI 665283	Col.#6197	USA, Colorado		4
117	PI 674265	Chucapaca	Bolivia, La Paz	Northern Highland	2

[a] Source of materials was determined from the USDA Germplasm Resources Information Network (GRIN). Asterisks indicate accessions donated to the USDA by Emigdio Ballón (Ballón collection).

[b] Ecotype group from UPGMA cluster analysis using allelic data from 36 microsatellite markers (Christensen et al. 2007).

[c] Genotypic group identified by hierarchical agglomerative clustering in the present study. G1: Peruvian and Bolivian highlands, G2: Bolivian highlands, G3: Ballón collection plus Bolivian highlands and G4: Ballón collection plus Sea level.
